# Dectin-1 aggravates neutrophil inflammation through caspase-11/4-mediated macrophage pyroptosis in asthma

**DOI:** 10.1186/s12931-024-02743-z

**Published:** 2024-03-08

**Authors:** Runjin Cai, Xiaoxiao Gong, Xiaozhao Li, Yuanyuan Jiang, Shuanglinzi Deng, Jiale Tang, Huan Ge, Chendong Wu, Huan Tang, Guo Wang, Lei Xie, Xuemei Chen, Xinyue Hu, Juntao Feng

**Affiliations:** 1 Department of Respiratory Medicine, National Key Clinical Specialty, Branch of National Clinical Research Center for Respiratory Disease, Xiangya Hospital, Central South University, Changsha, 410008 Hunan China; 2 Department of Nephrology, Xiangya Hospital, Central South University, Changsha, 410008 Hunan China; 3 National Clinical Research Center for Geriatric Disorders, Xiangya Hospital, Central South University, Changsha, 410008 Hunan China

**Keywords:** Asthma, Dectin-1, Caspase-11, Pyroptosis, Neutrophil

## Abstract

**Background:**

The pattern recognition receptor Dectin-1 was initially discovered to play a pivotal role in mediating pulmonary antifungal immunity and promoting neutrophil-driven inflammation. Recent studies have revealed that Dectin-1 is overexpressed in asthma, but the specific mechanism remains elusive. Additionally, Dectin-1 has been implicated in promoting pyroptosis, a hallmark of severe asthma airway inflammation. Nevertheless, the involvement of the non-classical pyroptosis signal caspase-11/4 and its upstream regulatory mechanisms in asthma has not been completely explored.

**Methods:**

House dust mite (HDM)-induced mice was treated with Dectin-1 agonist Curdlan, Dectin-1 inhibitor Laminarin, and caspase-11 inhibitor wedelolactone separately. Subsequently, inflammatory cells in bronchoalveolar lavage fluid (BALF) were analyzed. Western blotting was performed to measure the protein expression of caspase-11 and gasdermin D (GSDMD). Cell pyroptosis and the expression of chemokine were detected in vitro. The correlation between Dectin-1 expression, pyroptosis factors and neutrophils in the induced sputum of asthma patients was analyzed.

**Results:**

Curdlan appeared to exacerbate neutrophil airway inflammation in asthmatic mice, whereas wedelolactone effectively alleviated airway inflammation aggravated by Curdlan. Moreover, Curdlan enhanced the release of caspase-11 activation fragments and N-terminal fragments of gasdermin D (GSDMD-N) stimulated by HDM both in vivo or in vitro. In mouse alveolar macrophages (MH-S cells), Curdlan/HDM stimulation resulted in vacuolar degeneration and elevated lactate dehydrogenase (LDH) release. In addition, there was an upregulation of neutrophil chemokines CXCL1, CXCL3, CXCL5 and their receptor CXCR2, which was suppressed by wedelolactone. In asthma patients, a positive correlation was observed between the expression of Dectin-1 on macrophages and caspase-4 (the human homology of caspase-11), and the proportion of neutrophils in induced sputum.

**Conclusion:**

Dectin-1 activation in asthma induced caspase-11/4 mediated macrophage pyroptosis, which subsequently stimulated the secretion of chemokines, leading to the exacerbation of airway neutrophil inflammation.

**Graphical Abstract:**

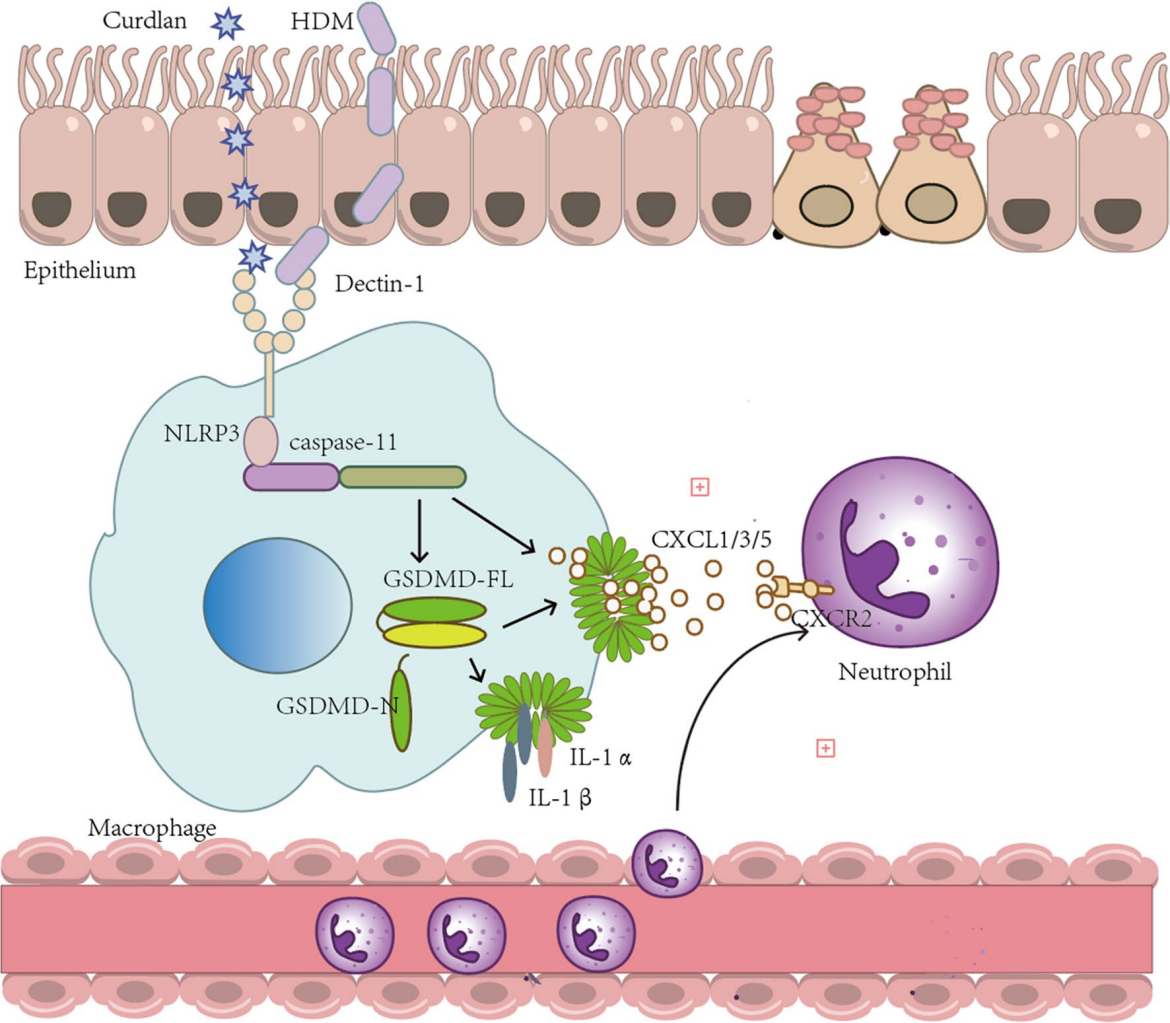

**Supplementary Information:**

The online version contains supplementary material available at 10.1186/s12931-024-02743-z.

## Introduction

Asthma is a chronic inflammatory airway disease marked by reversible airflow restriction and airway hyperresponsiveness [1]. Depending on the predominant infiltrating cells types in the lung, asthma can be categorized into distinct inflammatory endotypes [2–5]. Notably, a prominent airway neutrophil inflammation is often associated with the asthma severity and insensitivity to treatment with ICS [6, 7]. External factors like smoking, air pollution, and persistent infection with pathogenic microorganisms have been implicated in driving neutrophilic inflammation in asthma [8–11]. The underlying inflammatory mechanism of asthma aggravation caused by these exogenous factors are intricately tied to cell membrane receptors and the innate immune system. Pattern recognition receptors (PRRs), including toll-like receptors (TLRs) and so on, which mainly express on innate immune cells, play a pivotal role in recognizing external allergens [12], cigarette smoke [13], pathogenic microorganisms [14] and other stimuli. Subsequently, intracellular signaling cascades are activated, leading to production of pro-inflammatory mediators [15]. Therefore, targeting these innate receptors and inflammatory mediators emerges as a promising therapeutic strategy for neutrophilic asthma.

Dectin-1, a member of the C-type lectin receptor family as PRRs, has the capacity to recognize fungal β-glucan [16]. Its downstream signaling pathway involves the recruitment of caspase adaptor domain family member 9 (CARD9), which forms a signaling complexes with B cell CLL/lymphoma 10 (BCL10) and MALT1 paracaspase (MALT1). This cascade ultimately leads to neutrophilic inflammation mediated by T helper 1 (Th1), T helper 17 (Th17) cells or chemokine [17–20]. Of note, Dectin-1 expression is significantly higher in patients with severe asthma with fungal sensitization (SAFA) than patients with asthma alone [21, 22]. Combined exposure of house dust mite (HDM) and β-glucan has been reported to result in increased airway neutrophil infiltration and steroid resistance due to the up-regulation of Th17 cytokine [23, 24]. However, little is known about the precise correlation and underlying mechanisms between Dectin-1 and neutrophil infiltration in asthma.

Pyroptosis is a form of programmed cell death typically characterized by the cleavage of gasdermin D (GSDMD), resulting in the formation of pores in the cytoplasmic membrane [25]. Excessive pyroptosis has been shown to exacerbate airway inflammation and airway injury [26]. In severe steroid resistance and neutrophil asthma, there is an increase in the expression of pyroptosis-related inflammatory corpuscles, such as NOD-, LRR- and pyrin domain-containing protein 3 (NLRP3), as well as its downstream effector, caspase-1 [27, 28]. The cleavage of the gasdermin family member is primarily mediated by cysteine family. Specifically, caspase-11 has been identified as a key central molecule that cleave GSDMD [25]. Recent studies have found that caspase-11 deficiency mice exhibited a marked reduction in lung inflammation, highlighting the important role of this protease in the regulation of pyroptosis and associated inflammatory [29, 30]. In the mice model of *Aspergillus fumigatus* infection, it has been observed that both Dectin-1 and caspase-11 contribute to pulmonary neutrophil inflammation [31]. Additionally, Dectin-1 has been implicated in promoting microglial pyroptosis in neuroinflammation after cerebral hemorrhage [32]. Nevertheless, whether Dectin-1 can act as an upstream regulator of caspase-11, thereby facilitating pyroptosis in asthma remains unexplored.

In this study, we have demonstrated the expression of Dectin-1 in the macrophage of asthma patients and HDM-induced asthma mice. We have further investigated the underlying mechanism by which Dectin-1 mediated neutrophilic inflammation and lung damage. Our findings revealed that the macrophage-derived Dectin-1/caspase-11/GSDMD pathway exhibited a pivotal pathogenic role in asthma via enhancing neutrophil migration through CXCL1/CXCL3/CXCL5-CXCR2 axis.

## Materials and methods

### Human subjects

Subjects aged between 18 and 70 years with a diagnosis of asthma, based on the Global Initiative for Asthma (GINA2023 guidelines), were recruited from Xiangya Hospital in Changsha, Hunan province, China [33]. Exclusion criteria for subjects was applied: individuals were excluded if they (1) were receiving anti-asthma medications, (2) had other respiratory diseases including emphysema, bronchiectasis, pneumonia, tuberculosis, interstitial lung disease, chronic cough that cannot be attributed by asthma or chronic obstructive pulmonary disease, (3) were pregnant or lactating women, (4) had a serious non-lung disease, including heart failure, cancer, or serious mental illness. In addition, healthy individuals aged 18 years or older, without any respiratory ailments and with normal chest CT/X-ray and lung function tests, were contained from the hospital’s health examination center to serve as controls[34]. Comprehensive lung function and blood tests were conducted on all subjects. This study was ethically approved by the Medical Ethics Committee of Xiangya Hospital, Central South University (No.2022020475).

### Mice

Wild-type C57BL/6 mice used for asthma model were obtained from Hunan SJA Laboratory Animal Co. Ltd. Aged-matched female mice, ranging from six to eight weeks old, were held in specific-pathogen-free animal facilities with free access to water and food. All experimental procedures performed on animals followed the principles of Department of laboratory Animals of Central South University (No.2022020699).

### Mice model

Mice were treated with phosphate buffered solution (PBS; Servicebio, China), House dust mite (HDM; Greer Laboratories, Lenoir, NC, USA), Curdlan (Invivogen, USA), Laminarin (Solarbio Life Sciences, China), wedelolactone (Wed; Topscience, China) or lipopolysaccharide (LPS; Solarbio Life Sciences, China). To induce allergic airway inflammation, mice were firstly sedated using isoflurane for intratracheal administration. Subsequently, on days 0, 2, and 4, they were sensitized by HDM (20 μg HDM in 50 μl PBS) or PBS (50 μl) with or without Curdlan (20 μg Curdlan in 50 μl PBS) or Laminarin (5 mg/kg, 100 μl). The challenge phase was developed with the same dose of the sensitization stage at days 11 to 14. On day 8, an additional Curdlan or Laminarin interventions was administrated. Laminarin was given by intraperitoneal injection, while Curdlan was administrated intratracheally to mice prior to HDM or PBS exposure. For treatment with wedelolactone during sensitization and effector phase, mice were given wedelolactone (20 mg/kg, 200 μl) intragastric administration before stimulation with HDM or HDM/Curdlan.

The neutrophilic asthma model induced by the combination of HDM and LPS followed the protocol outlined previously [35]. Briefly, mice were received an intratracheal instillation of 20 μg HDM and 1 μg LPS suspended in 50 μl PBS for three days (days 0, 2, 4). Following a four-day break, the mice were then administrated only HDM (20 μg HDM in 50 μl PBS) intratracheally once daily for 4 days (9-12). On days 0, 2, 4, 9-12, the Laminarin or wedelolactone was given before stimulation with HDM or HDM/LPS.

One day after the final challenge, the mice were euthanized, BALFs were collected through two consecutive flushes with 0.8 ml PBS and centrifuged for 5 min at 5000 rpm and 4 ℃. The suspend precipitated cell with 300 μl PBS for subsequent flow cytometry. The supernatant of BALFs was stored at – 80 ℃. Additionally, lung tissues were washed by PBS, and the left of which were fixed in 4% paraformaldehyde for histological examination. The remaining lung tissues were stored at -80℃ for RNA and protein analysis.

### Flow cytometry and antibodies

Total cell counts were counted with 100 μl re-suspension of BALFs. The remaining resuspended cells underwent erythrocytes lysis using ACK lysis buffer, followed by staining with surface marker antibodies for 45 min avoiding light, and additionally, incubated with anti-Mouse CD16/CD32 (BD Biosciences) for 20 min.

The following antibodies were used to stain mouse cells: APC/Cyanine7 anti-mouse CD45 (BD Biosciences, Franklin Lakes, NJ, USA), PerCP/Cyanine5.5 anti-mouse Gr-1 (Biolegend), PE/Cyanine7 anti-mouse CD11c (BD Biosciences, Franklin Lakes, NJ, USA), Alexa Fluor® 488 anti-mouse CD11b (Biolegend), APC anti-mouse Siglec F (Biolegend), BV 421 anti-mouse CD3e (BD Biosciences, Franklin Lakes, NJ, USA), PE anti-mouse Dectin-1 (BD Biosciences, Franklin Lakes, NJ, USA). The following antibodies were used to stain human cells: PerCP/ Cyanine5.5 anti-human CD369 (Dectin-1; BD Biosciences, Franklin Lakes, NJ, USA), APC/Cyanine7 anti-human CD45 (BD Biosciences), BV421 anti-human CD68 (BD Biosciences, Franklin Lakes, NJ, USA), FITC anti-human CD16 (BD Biosciences, Franklin Lakes, NJ, USA), Alexa Fluor® 647 anti-human CD66b (BD Biosciences, Franklin Lakes, NJ, USA). For cells from mice BALFs, eosinophils were defined as CD11C^−^Siglec F^+^ cells. Macrophages were identified as CD11C^+^Siglet F^+^ cells. Neutrophils were defined as FSC^high^SSC^high^Gr1^+^ cells. Lymphocytes were defined as FSC^high^SSC^high^CD3^+^ cells. For cells from induced sputum, eosinophils were defined as CD45^+^CD16^−^CD66b^+^ cells. Macrophages were identified as CD45^+^CD68^+^ cells. Neutrophils were defined as CD45^+^CD16^+^CD66b^+^ cells. Flow cytometric analysis were performed on a BD FACSCalibur (Becton, Dickinson and Company). The data was analyzed with FlowJo^TM^ v10.8.1 software (BD Life Sciences).

### Histology

The left lung tissue, harvested from mice after perfusion with PBS, was fixed with 4% paraformaldehyde (ECOTOP, China) for 48 h. Following this, the tissues were embedded in paraffin, sectioned to 3 μm, and subjected to staine with hematoxylin and eosin (H&E; ECOTOP, Chnia) and periodic acid-Schiff (PAS; Servicebio, China) for detailed analysis. To assess the degree of lung damage, an inflammation score was calculated as previously described [36]. In brief, the score of perivascular and peribronchiolar inflammation was determined as follows: a score of 0 indicated normal tissues; 1 denoted the presence of few cells; 2 represented a ring of inflammatory cells one cell layer thick; 3 signified a ring of inflammatory cells two to four cells thick; 4 indicated a ring of inflammatory cells of more than four cells thick. As for the evaluation of PAS-positive goblet cells in airway, a relative scoring system was utilized: a score of 0 represented less than 5% positive cells; 1 displayed 5–25% positivity; 2 showed 25–50% positivity; 3 denoted 50–75% positivity; and 4 signified more than 75% positivity.

### Cell culture and cell death assay

Mouse alveolar macrophages (MH-S cells) were cultured in RPMI-1640 medium (Gibco, US), supplemented with 10% fetal bovine serum (FBS; Gibco) and 1% penicillin–Streptomycin Solution (Gibco) at 37℃ in a humidified incubator containing 5% CO_2_.

Cell death was probed by LDH Cytotoxicity Assay kit (Nanjing Jiancheng Bioengineering Institute, China) according to the manufacturer’s instructions.

### Cell fluorescence

MH-S cell were placed on slides, fixed with 4% paraformaldehyde at room temperature for 30 min, then washed with PBS for 2–3 times. Placed the cell slides in 1% Triton x-100 (JISSKANG, China) for 10 min. After washing with PBS, the cell slides were blocked in 5% BSA for 1 h. Then the diluted anti-mouse caspase-11(ab240991, abcam, 1:100) was added and incubated overnight at 4 °C. On the second day, incubate the cell slides with the fluorescent secondary antibody (Beyotime, China) at 37 °C for 1 h under the dark environment. After rinsing, fluormount with DAPI (AntGene, China) was added to stain the nucleus. Finally, seal the tablet and observe the slides under the fluorescence microscope.

### Induction sputum

Sputum induction was carried out as previously described [37]. Briefly, participants inhaled a 3% hypertonic saline solution through a nebulizer for 20 min. Prior to coughing up sputum at two-minute intervals, participants were requested to remove any saliva from the mouth. Induced sputum sample was processed with the same volume of dithiothreitol (DTT) and bathed at 37 ℃ for 15 min. Dispersed sputum sample was then centrifuged at 1500 rpm for 5 min. The obtained cell precipitate was divided into two parts. One part was prepared into single cell suspension for flow cytometry and the other part was lysed by AG RNAex Pro Regent for RNA analysis.

### Real-time quantitative PCR analysis

RNA was extracted by AG RNAex Pro Regent (Accurate Biotechnology, Hunan, China). cDNA synthesis was performed with 2 × Hieff® PCR Master Mix (Yeasen Biotechnology, Shanghai, China). Quantitative RT-PCR was performed with Hieff® qPCR SYBR Green Master Mix (Yeasen Biotechnology, Shanghai, China). The relative interest mRNA expression was normalized to GAPDH using the ΔΔCt method. The primer sequences are shown in Table 1.

**Table 1:** Sequences of gene for qPCR

	Forward primer	Reverse primer
Caspase-11 (mouse)	CCTGAAGAGTTCACAAGGCTT	CCTTTCGTGTAGGGCCATTG
IL-1α (mouse)	AGTATCAGCAACGTCAAGCAA	TCCAGATCATGGGTTATGGACTG
IL-1β (mouse)	GAAATGCCACCTTTTGACAGTG	TGGATGCTCTCATCAGGACAG
IL-6 (mouse)	TAGTCCTTCCTACCCCAATTTCC	TTGGTCCTTAGCCACTCCTTC
IL-17a (mouse)	TTTAACTCCCTTGGCGCAAAA	CTTTCCCTCCGCATTGACAC
CXCR2 (mouse)	ATGCCCTCTATTCTGCCAGAT	GTGCTCCGGTTGTATAAGATGAC
iNOS (mouse)	AATCTTGGAGCGAGTTGTGG	CAGGAAGTAGGTGAGGGCTTG
TNF-α (mouse)	CAGGCGGTGCCTATGTCTC	CGATCACCCCGAAGTTCAGTAG
Arg1 (mouse)	CTCCAAGCCAAAGTCCTTAGAG	GGAGCTGTCATTAGGGACATCA
Fizz1 (mouse)	CCAATCCAGCTAACTATCCCTCC	CCAGTCAACGAGTAAGCACAG
Dectin-1 (mouse)	CAGGTCTGGCAATTCTTCTGAA	GTCTTGCTCATGTGTGTAAGTGA
CXCL1 (mouse)	CCACACTCAAGAATGGTCGC	TCTCCGTTACTTGGGGACAC
CXCL3 (mouse)	TGAGACCATCCAGAGCTTGACG	CCTTGGGGGTTGAGGCAAACTT
CXCL5 (mouse)	GTTCCATCTCGCCATTCATGC	GCGGCTATGACTGAGGAAGG
CXCL2 (mouse)	CCAACCAGGCTACAGG	GCGTCACACTCAAGCTCTG
CXCL15 (mouse)	TCGAGACCATTTACTGCAACAG	CATTGCCGGTGGAAATTCCTT
G-CSF (mouse)	GGTGTCCTGGCCATTTCGTA	TCTGTCCTCTCCTACCACGC
Caspase-4 (human)	CTCGAGGCCCCTGGGGCAGAAGCCTC	GATATCGGGGCTCAGCAGGCGGGTCT
IL-1α (human)	TGGTAGTAGCAACCAACGGGA	ACTTTGATTGAGGGCGTCATTC
IL-1β (human)	AGCTACGAATCTCCGACCAC	CGTTATCCCATGTGTCGAAGAA
IL-18 (human)	TGCTACACCTTCCTCCTGCT	TATGATGCTGGGGAGAGACC

### Western blotting

Proteins of cells and lung tissues were extracted by RIPA Lysis Buffer (CoWin Biosciences, China) with phenylmethanesulfonylfluoride (PMSF; 1:100, MilliporeSigma) and protease inhibitor cocktail (1:100, Servicebio, China) and quantified by BCA protein assay kit (ECOTOP, China) for SDS-PAGE electrophoresis. Separated proteins were transferred to a polyvinylidene difluoride membrane (PVDF). The PVDF membrane was incubated with first antibodies overnight at 4 ℃ after blocked with 5% skim milk for 1 h. Antibodies against caspase-11 (ab240991, abcam, 1:1000), Gsdmd (ab219800, abcam, 1:1000), NLRP3 (15101, Cell Signaling Technology, 1:1000) and HRP-conjugated secondary antibodies (Proteintech, 1: 10,000) were used.

### Statistics analysis

Results are expressed as mean ± SD or mean ± SEM. Statistical differences were calculated by unpaired Student’s t test between two groups. One-way ANOVA was used to evaluated multiple groups. For non-normal distribution data, Mann–Whitney U test or non-parametric one-way ANOVA was carried out. Correlations were evaluated by Pearson’s rank correlation or Spearman rank correlation. All data were calculated by GraphPad Prism (version 9.0 GraphPad Software, San Diego California USA). A p-value less than 0.05 was defined as statistically significant.

## Results

### Dectin-1 activation predominantly enhances pulmonary neutrophil inflammation in asthmatic mice

**Fig. 1:**
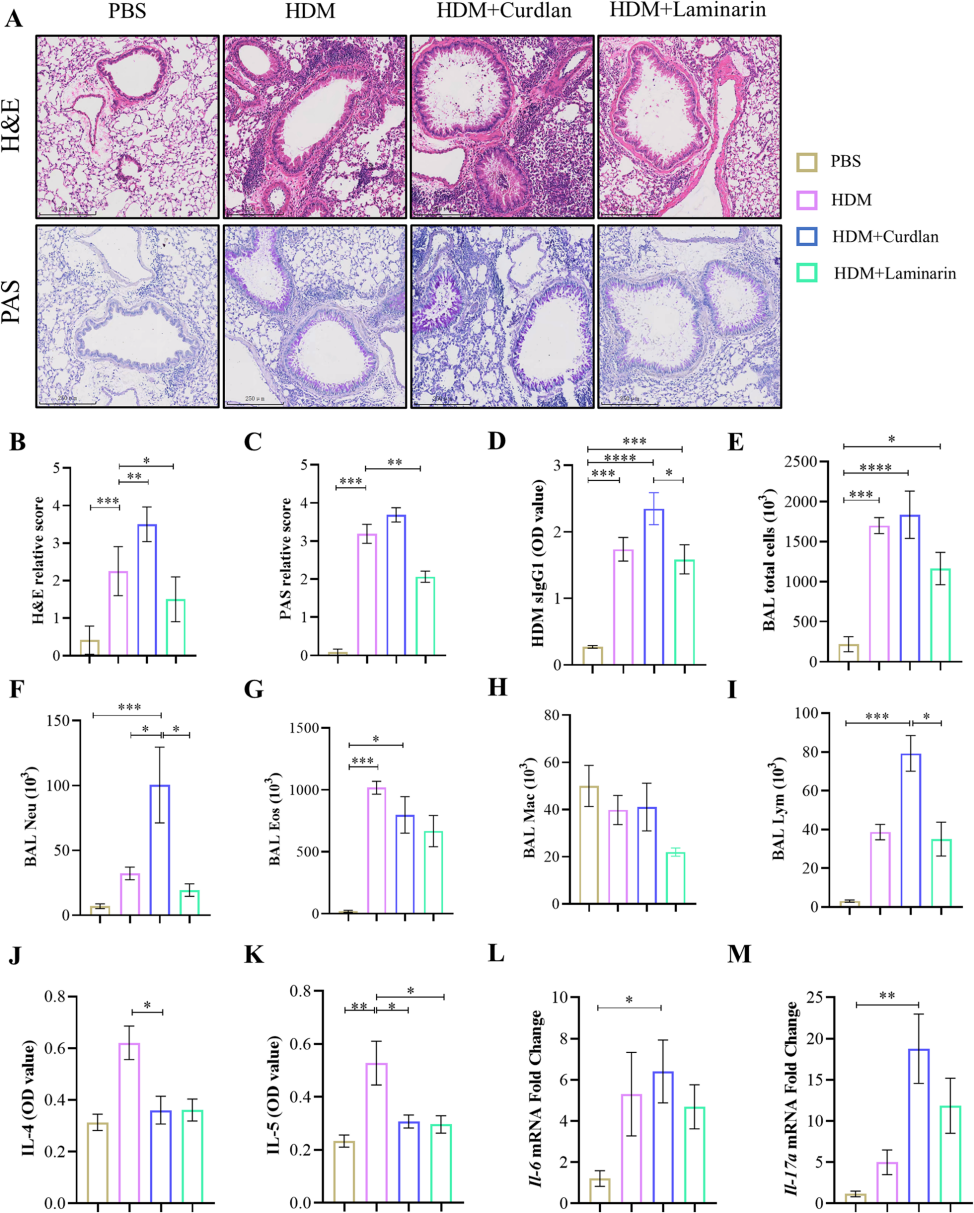
Curdlan mainly enhances pulmonary neutrophil inflammation in asthmatic mice. C57BL/6 mice were randomly divided into 4 groups, PBS, HDM, HDM + Curdlan and HDM + Laminarin group. Mice received either Curdlan (20 μg Curdlan in 50 μl PBS) or Laminarin (5 mg/kg, 100 μl) prior to HDM at days 0, 2, 4, 11 to 14. **A** The lung tissue of each group was stained with H&E and PAS (magnification: 25x). **B**, **C** Histopathological inflammatory score were assessed based on H&E staining and PAS staining. **D** Levels of HDM specific IgG1 (sIgG1) in BALF were quantitated using a mouse specific ELISA kit. **E–I** The counts of total cells and neutrophils, eosinophils, macrophages and lymphocytes in BALF of each mice group were detected by flow cytometry. **J**, **K** Detection of *IL-4* and *IL-5* cytokines in BALF of mice in each group by ELISA kit. **L-M.** mRNA expression of *IL-6* and *IL-17a* in the lungs of mice measured using qRT-PCR (n = 6–8) (*P < 0.05; **P < 0.01;***P < 0.005; ****P < 0.001, one way ANOVA, error bars represent mean ± SEM)

To elucidate the general role of Dectin-1 in asthma, Dectin-1 agonist Curdlan and inhibitor Laminarin was used to treat HDM-induced asthma mice. As assessed by H&E and PAS staining, Curdlan aggravated lung tissues damage, increased infiltrating inflammatory cells in blood vessels and peribronchial, thickened alveolar wall and enhanced mucus production, while Laminarin existed opposite phenomenon (Fig. 1A–C). Consistent with histologic observation, HDM specific IgG1 (Fig. 1D), total cells (Fig. 1E) and neutrophils (Fig. 1F) in BALF were significantly increased in Curdlan-treated asthmatic mice but decreased in Laminarin-treated asthmatic mice. However, there were no significant changes of eosinophils, macrophages and lymphocytes (Fig. 1G–I). Moreover, while Curdlan had minimal impact on the level of IL-4 and IL-5 in BALF (Fig. 1J, K), it did upregulate the transcription level of *IL-6* and *IL-17a* (Fig. 1L, M). Additionally, to verify the role of Dectin-1 in normal mice, mice were treated with Curdlan and Laminarin alone. Curdlan alone could cause mild lung inflammation, whereas no significant changes were observed in Laminarin-treated group (Additional file 1: Fig. S1). Collectively, these findings suggest that the activation of Dectin-1 aggravates pulmonary neutrophil inflammation in asthma.

### Caspase-11 and pyroptosis is activated in Dectin-1 agonist induced asthma mice

**Fig. 2:**
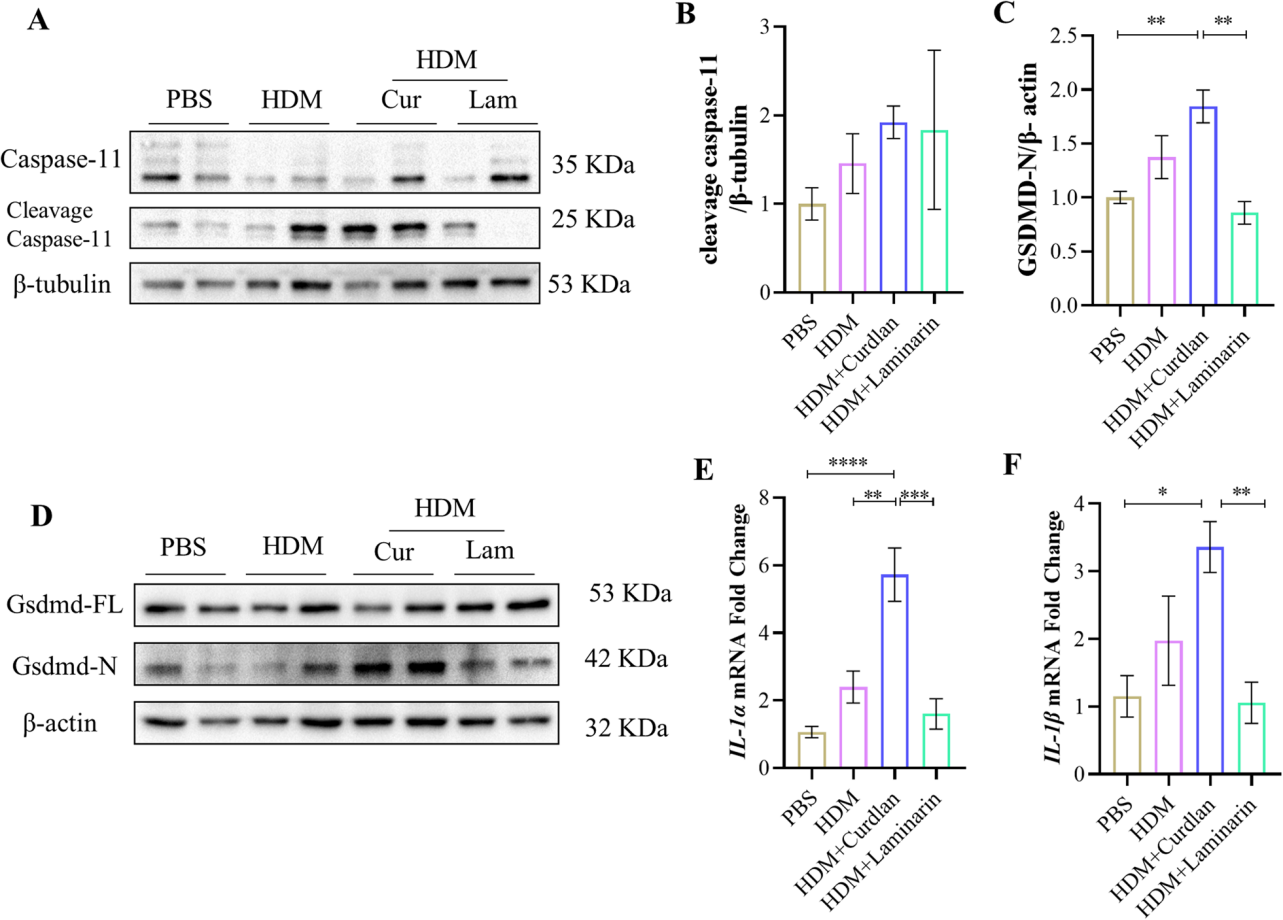
Caspase-11 and pyroptosis is activated in Dectin-1 agonist induced asthma mice. **A** Expression of pro caspase-11 and cleavage caspase-11 in mice lung tissue by western blot. **B** Densitometric quantification of immunoblots in (**A**). Levels of cleavage caspase-11 were normalized to β-tubulin. **C** Densitometric quantification of immunoblots in **(D). D **Expression of full length of gasdermin D (GSDMD-FL) and N-terminal fragments of gasdermin D (GSDMD-N) in mice lung tissue by western blot. **B** Levels of GSDMD-N were normalized to β-actin. **E**, **F** *IL-1α* and *IL-1β* mRNA expression in lung tissue of mice by qRT-PCR. (n = 4–6). (*P < 0.05; **P < 0.01; ***P < 0.005; ****P < 0.001, one way ANOVA, error bars represent mean ± SEM)

Fluorescence co-localization in lung tissues revealed an increased expression of caspase-11 in asthmatic mice treated with Curdlan (Additional file 1: Fig. S3). Therefore, we investigated whether Curdlan activates caspase-11-related pyroptosis pathway in asthma. Western blotting showed that caspase-11 release activated cleavage fragments and GSDMD was cleaved to expose the N-terminal in HDM-induced mice. This phenomenon was more pronounced in lung tissues of Curdlan-induced asthmatic mice, while with downregulated tendency in Laminarin treated group (Fig. 2A–D). Additionally, the transcription level of *IL-1α* and *IL-1β* in lung tissue of asthmatic mice were also elevated following Curdlan treatment (Fig. 2E, F). These findings suggest that Dectin-1 activation in HDM-induced asthmatic mice may interfere with the process of pyroptosis by promoting caspase-11 activation and GSDMD cleavage.

### Caspase-11 inhibition alleviates neutrophilic predominant asthma

**Fig. 3:**
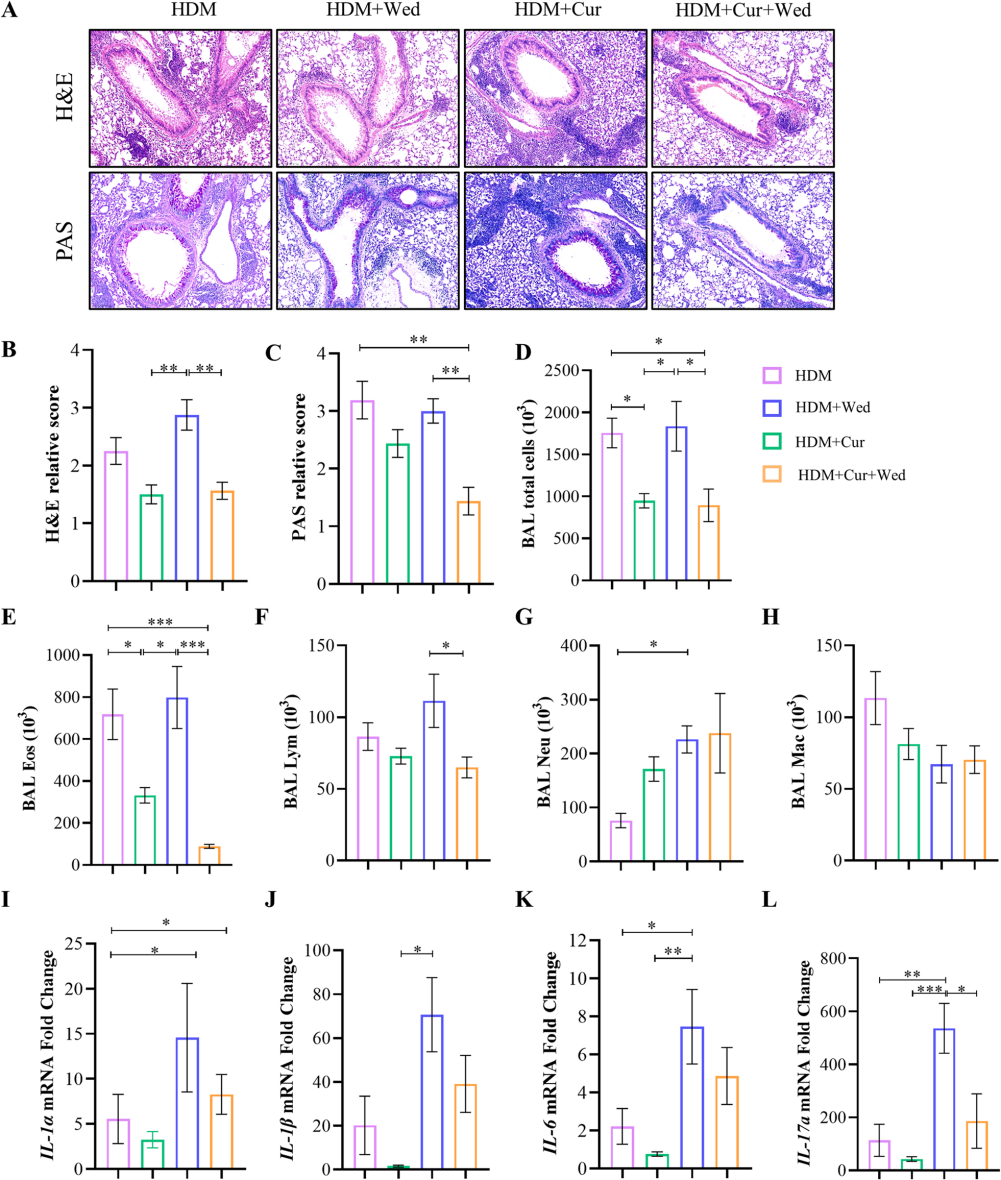
Caspase-11 inhibitor has therapeutic effect on Curdlan-induced asthma mice. C57BL/6 mice were divided into 4 groups, HDM, HDM + Wed (wedelolactone), HDM + Cur (Curdlan) and HDM + Cur + Wed group. Wedelolactone (20 mg/kg, 200 μl) was administered to mice before stimulation with HDM or HDM/Curdlan at days 0, 2, 4, 11 to 14. **A** The lung tissue of each group was stained with H&E and PAS (25x). **B**, **C** Inflammatory score of lung histopathology by H&E staining and PAS staining. **D**–**H** The counts of total cells and eosinophils, lymphocytes, neutrophils and macrophages and in BALF of each mice group were detected by flow cytometry. **I–L** mRNA expression of *IL-1α, IL-1β, IL-6* and *IL-17a* in the lungs of mice by qRT-PCR. (n = 4–8). (*P < 0.05; **P < 0.01;***P < 0.005; ****P < 0.001, one way ANOVA, error bars represent mean ± SEM) Wed: wedelolactone

To verify whether Dectin-1 contributes to asthma pathogenesis through caspase-11 induced pyroptosis, we utilized the caspase-11 specific inhibitors wedelolactone, derived from eclipta prostrata L, in asthma mice with or without Curdlan induction. Surprisingly, wedelolactone reduced peribronchial and perivascular inflammation, goblet cells and mucus production in HDM-induced asthmatic mice. Notably, its inhibitory effects were more pronounced in Curdlan-treated asthmatic mice (Fig. 3A–C). What’s more, wedelolactone administration decreased BALF total cells and eosinophils, lymphocytes in asthma mice, especially in those treated with Curdlan (Fig. 3D–F). However, the presence or absence of wedelolactone was not significantly associated with BALF neutrophils and macrophages (Fig. 3G, H). Additionally, wedelolactone inhibited the *IL-1α, IL-1β*, *IL-6* and *IL-17a* mRNA expression, which were related to neutrophilic predominant asthma (Fig. 3I–L).

It is well-known that LPS can aggravate the airway neutrophil inflammation in asthma mice. Thus, we used Laminarin and wedelolactone in HDM/LPS-induced mice to validate their therapeutic effect on neutrophilic asthma. First, flow cytometry showed that the expression of Dectin-1 in HDM/LPS asthma was higher, although the difference was not statistically significant (Additional file 1: Fig. S2A). H&E and PAS staining analysis displayed that inflammation and mucus production in HDM/LPS exposure lung tissues were alleviated after wedelolactone treatment, while Laminarin had no significant effect (Additional file 1: Fig. S2B–D). Additionally, wedelolactone also suppressed BALF total cell count (Additional file 1: Fig. S2E), eosinophil (Additional file 1: Fig. S2F) and lymphocyte count (Additional file 1: Fig. S2G) in HDM/LPS stimulated asthma mouse model. Moreover, wedelolactone also inhibited neutrophils in BALF, this effect was not statistically significant (Additional file 1: Fig. S2H). Taken together, these findings suggest that wedelolactone has the potential to reduce the airway inflammation of neutrophilic predominant asthma.

### Activation of Dectin-1 augments pyroptosis and pro-inflammatory response in MH-S cells

**Fig. 4:**
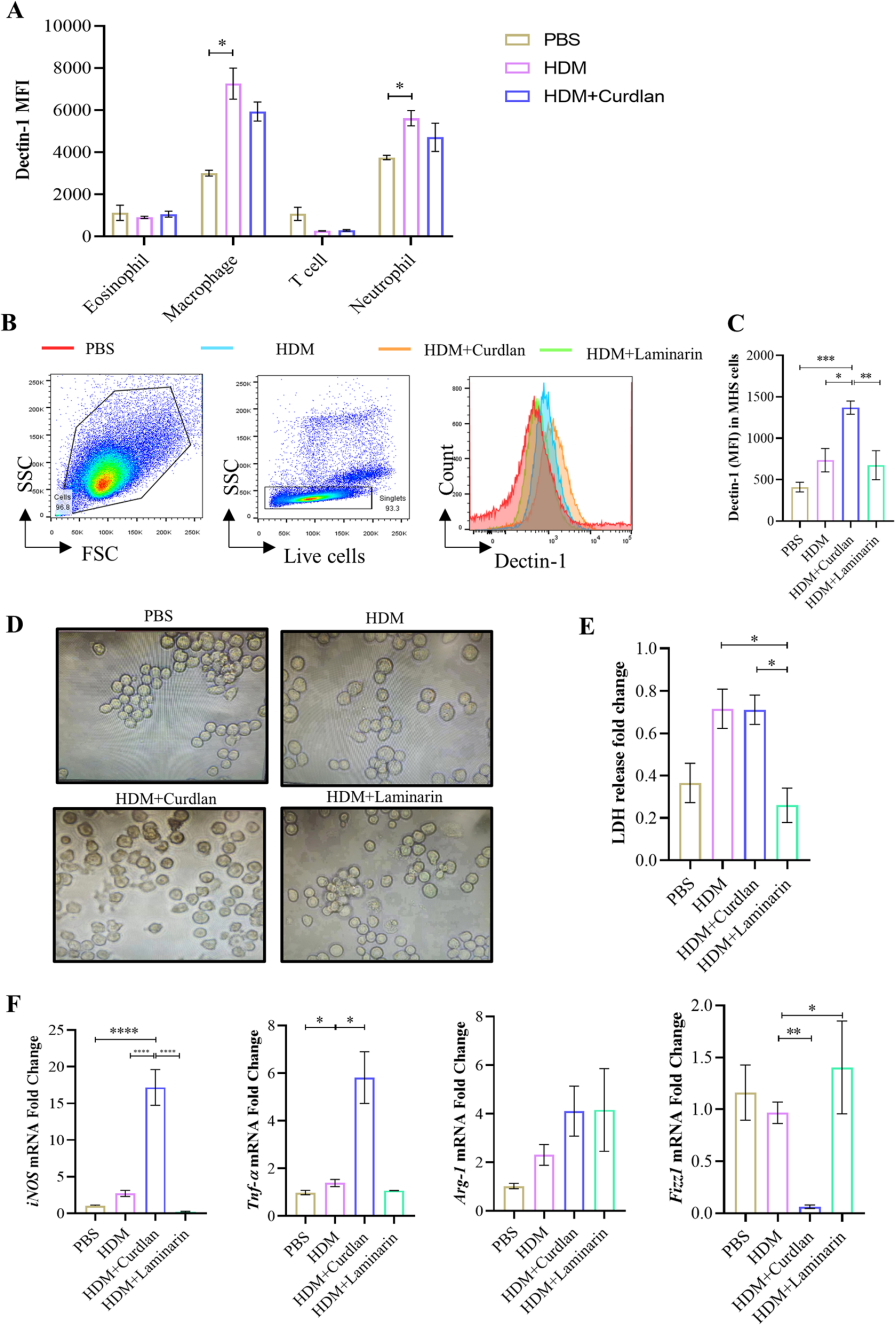
Activation of Dectin-1 promotes pyroptosis and pro-inflammatory function in MH-S cells. **A** The mean fluorescence intensity (MFI) of Dectin-1 on inflammatory cells in BALF of mice in each group by flow cytometry. MH-S cells were cultured in vitro, stimulated with PBS (50 μg/ml), HDM (50 μg/ml), HDM plus Curdlan (200 μg/ml) or HDM plus Laminarin(1 μg/ml) for 24 h. **B**, **C** The MFI of Dectin-1 expression on MH-S cells in each stimulation group by flow cytometry. **D** Optical microscope images of cell morphology in each stimulation group. **E** LDH release in supernatant of cells in each stimulation group. **F** The mRNA expression level of iNOS, TNF-α, Arg-1, Fizz1 in each stimulation group by qRT-PCR. (*P < 0.05; **P < 0.01;***P < 0.005; ****P < 0.001, one way ANOVA, error bars represent mean ± SEM)

In vivo investigations utilizing flow cytometry showed that Dectin-1 was mainly expressed on macrophages in BALF of asthmatic mice (Fig. 4A). To further validate the effect of Dectin-1 on macrophage functionality and pyroptosis in vitro, MH-S cells were stimulated with HDM followed by Curdlan or Laminarin for 24 h. Subsequently, the expression of Dectin-1 on MH-S cells was analyzed (Fig. 4B and C).

Upon HDM stimulation, MH-S cells exhibited slight swelling and deformation. However, when cells were co-treated with HDM and Curdlan, marked plasma membrane blistering and pyroptosis became evident. Notably, in the presence of an inhibitor, cell pyroptosis was absent (Fig. 4D). Additionally, LDH release, a marker of cell membrane damage, was increased in HDM alone group and HDM plus Curdlan group, while significantly decreased in Laminarin-treated HDM group (Fig. 4E). Moreover, our observation highlighted that Curdlan mainly selectively increased the transcription level of pro-inflammatory cytokines inducible nitric oxide synthase (iNOS) and tumour necrosis factor (TNF-α) (Fig. 4F). Taken together, these data indicate macrophage Dectin-1 may play a pivotal role in asthma pathogenesis via modulating pyroptosis and pro-inflammatory response.

### Curdlan augments while wedelolactone suppresses caspase-11 activation and GSDMD cleavage in HDM-induced MH-S cells

**Fig. 5:**
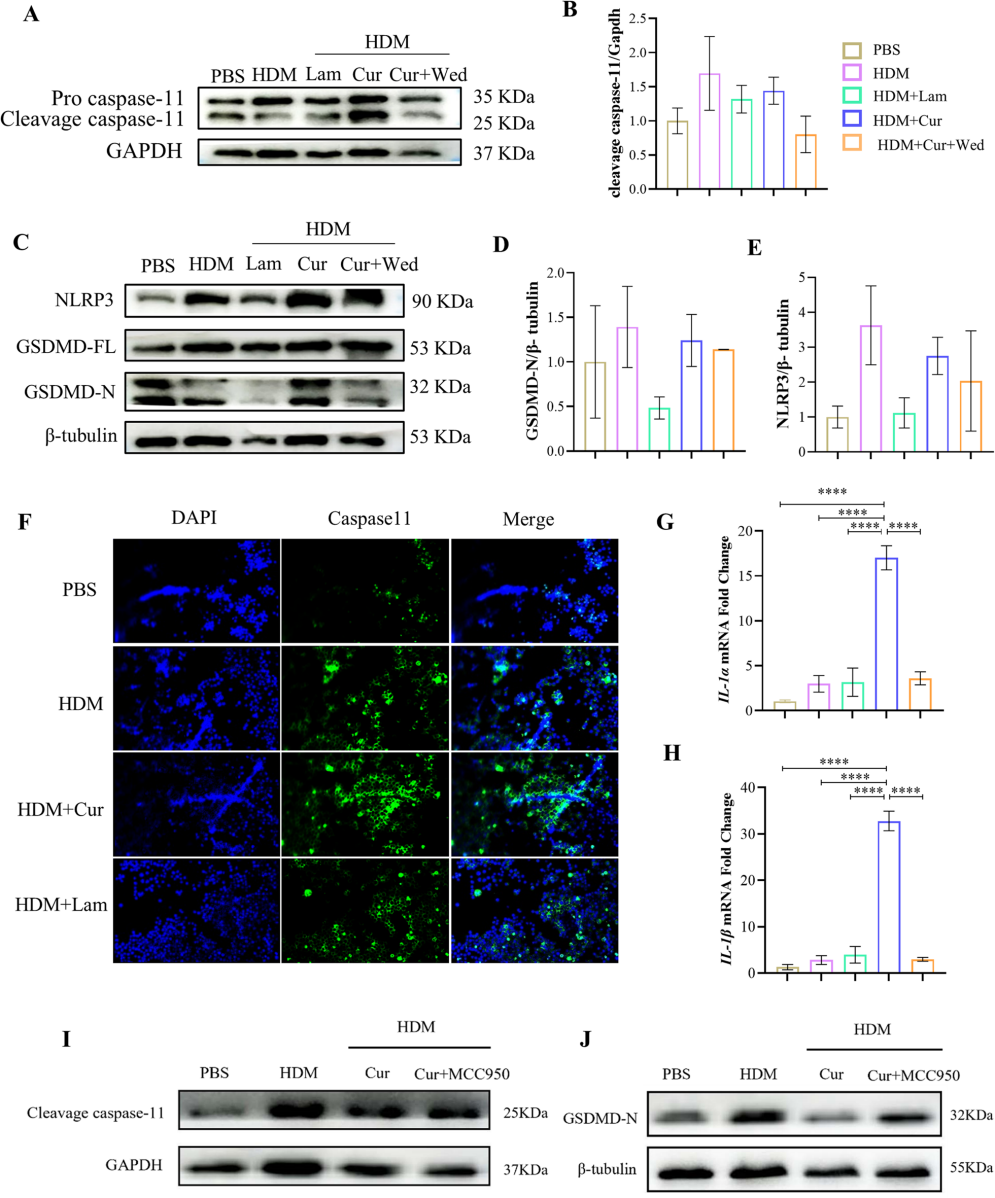
Curdlan increases but wedelolactone downregulates caspase-11 activation and GSDMD cleavage in HDM-induced MH-S cells. MH-S cells were stimulated with PBS (50 μg/ml), HDM (50 μg/ml), HDM plus Laminarin (Lam) (1 μg/ml), HDM plus Curdlan (Cur) (200 μg/ml), or giving wedelolactone (Wed) (30 μM) after HDM/Curdlan for 24 h. **A** Total proteins were extracted and subjected, western blot was used to detect the expression of pro caspase-11 and cleavage caspase-11 in each stimulation group. **B** Densitometric quantification of immunoblots in (**A**). Levels of cleavage caspase-11 were normalized to GAPDH. **C** Expression of GSDMD-FL, GSDMD-N and NLRP3 in each stimulation group by western blot. **D** Densitometric quantification of immunoblots in (**C**). Levels of GSDMD-N were normalized to β-tubulin. **E** Densitometric quantification of immunoblots in **(C).** Levels of NLRP3 were normalized to β-tubulin. **F** Representative images of caspase-11 expression in each stimulation group detected by immune fluorescence microscopy. **G**, **H** The expression of *IL-1α* and *IL-1β* mRNA in the cells of each stimulation group by qRT-PCR. MH-S cells were stimulated with PBS (50 μg/ml), HDM (50 μg/ml), HDM plus Curdlan (200 μg/ml), or giving MCC950(10 μM) after HDM/Curdlan for 24 h, cleavage caspase-11 (**I**) and GSDMD-N (**J**) in each stimulation group by western blot. (*P < 0.05; **P < 0.01; ***P < 0.005; ****P < 0.001, one way ANOVA, error bars represent mean ± SEM)

Fluorescence co-localization analysis of lung tissues revealed that caspase-11 was also predominantly expressed in macrophages (Fig. S3). Thus, we next determined whether the macrophage Dectin-1-regulated pyroptosis is mediated by caspase-11 in vitro. Western blot detection showed that HDM stimulation led to increased caspase-11 activation (Fig. 5A and B) and GSDMD cleavage (Fig. 5C and D), which were inhibited by Laminarin and enhanced by Curdlan, respectively. In the presence of HDM and Curdlan stimulation, the inhibition of caspase-11 (Fig. 5A and B) reduced the N-terminal exposure of GSDMD (Fig. 5C and D). Cell immunofluorescence showed that Curdlan stimulation was able to exacerbate HDM-induced intracellular caspase-11 expression (Fig. 5F). The expression of *IL-1α* (Fig. 5G) and *IL-1β* (Fig. 5H) mRNA elevated after HDM/Curdlan stimulation, an effect that was inhibited by wedelolactone. These findings suggest that Dectin-1-mediated pyroptosis is related to caspase-11-GSDMD axis.

Dectin-1 has been established as an important initiator of NLRP3 inflammasome [38, 39]. NLRP3 can also activate caspase-11 through independent pathway [40]. In line with this, our study also found the upregulation of NLRP3 expression after HDM/Curdlan stimulation, which was inhibited after HDM/Laminarin stimulation (Fig. 5C and E). When NLRP3 inhibitor MCC950 was added to the cells stimulated with HDM plus Curdlan, both the activation of caspase-11 (Fig. 5I) and GSDMD cleavage (Fig. 5J) were inhibited. The results implicate that the Dectin-1 activated caspase-11 may be mediated by NLRP3.

### Curdlan increase but wedelolactone downregulate neutrophil related chemokine in HDM-induced MH-S cells

**Fig. 6:**
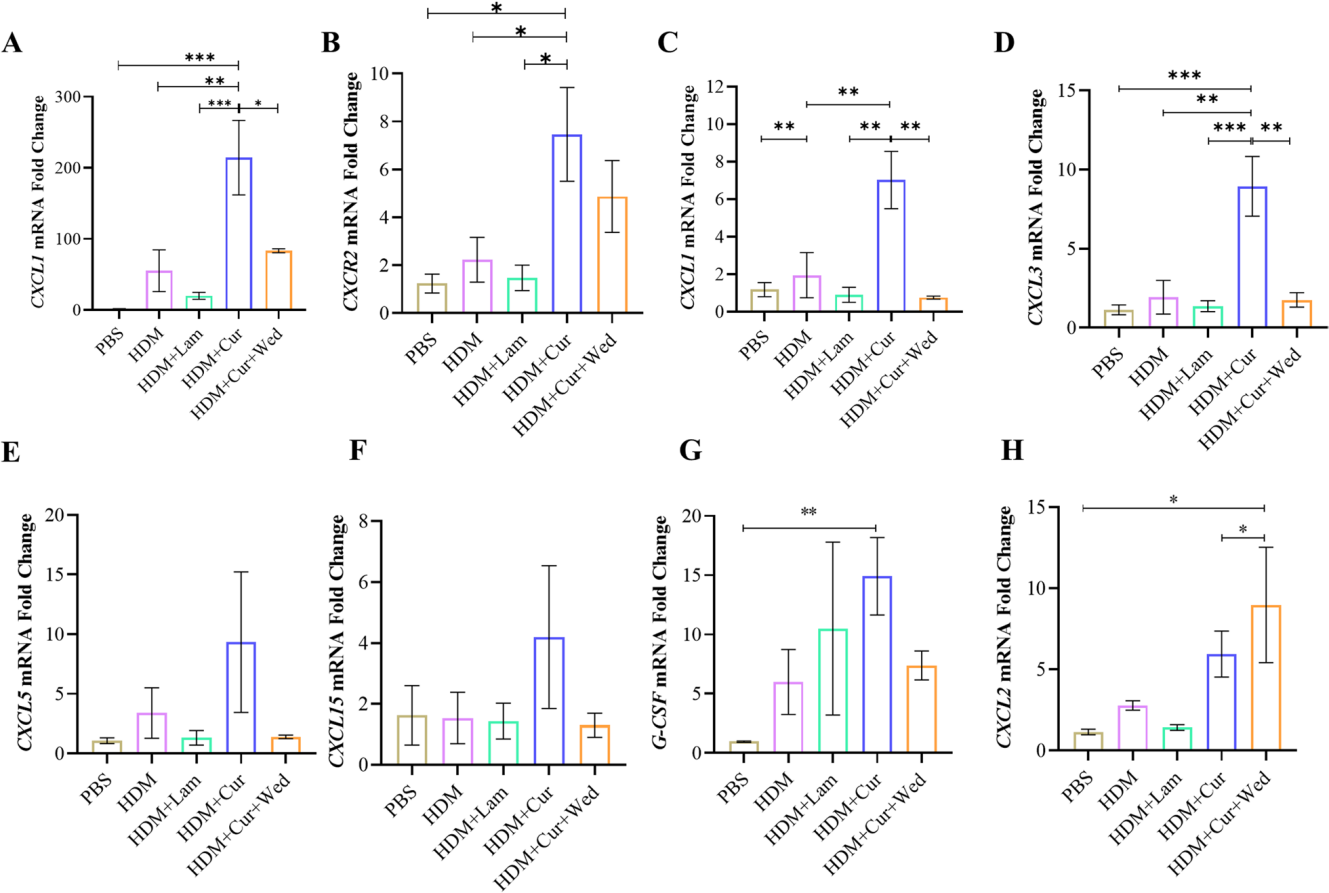
Curdlan increase but wedelolactone downregulate neutrophil related chemokine CXCL1 in HDM-induced MH-S cells. **A**, **B** mRNA expression of CXCL1and CXCR2 in the lungs of mice by qRT-PCR. MH-S cells were stimulated with PBS (50 μg/ml), HDM (50 μg/ml), HDM plus Laminarin (Lam)(1 μg/ml), HDM plus Curdlan (Cur) (200 μg/ml), or giving wedelolactone (Wed) (30 μM) after HDM/Curdlan for 24 h. CXCL1 (**C**), CXCL3 (**D**), CXCL5 (**E**), CXCL15 (**F**), G-CSF (**G**) and CXCL2 (**H**) mRNA expression levels in each group by qRT-PCR. (*P < 0.05; **P < 0.01;***P < 0.005; ****P < 0.001, one way ANOVA, error bars represent mean ± SEM)

In order to elucidate the mechanism by which the macrophage Dectin-1/caspase-11 axis potentiates neutrophil inflammation, we examined the expression patterns of key neutrophil chemokines. It is well-known that the CXCL1-CXCR2 axis is related to neutrophil migration [41]. In the lung of HDM-induced mice, we observed an elevation in CXCL1 expression following Curdlan treatment, whereas wedelolactone administration led to a reduction. Moreover, the expression trend of CXCR2 was consistent with that of CXCL1 (Fig. 6A and B).

In vitro studies further corroborated these findings. Curdlan also apparently increased CXCL1 expression induced by HDM, but wedelolactone treatment existed decrease tendency (Fig. 6C). Similarly, Curdlan-induced CXCL3 expression was attenuated by wedelolactone (Fig. 6D), as well as the case for CXCL5, albeit without statistical significance (Fig. 6E). There was no significant effect on the expression of chemokine CXCL15, CXCL2 and G-CSF in different treated group (Fig. 6F–H). Therefore, the results suggest that the macrophage Dectin-1 mediated caspase-11/pyroptosis axis mainly affects the up-regulated expression of chemokine CXCL1/3/5, thus promoting neutrophil aggregation.

### Positive correlation between macrophage Dectin-1 and neutrophils in asthma patients

**Fig. 7:**
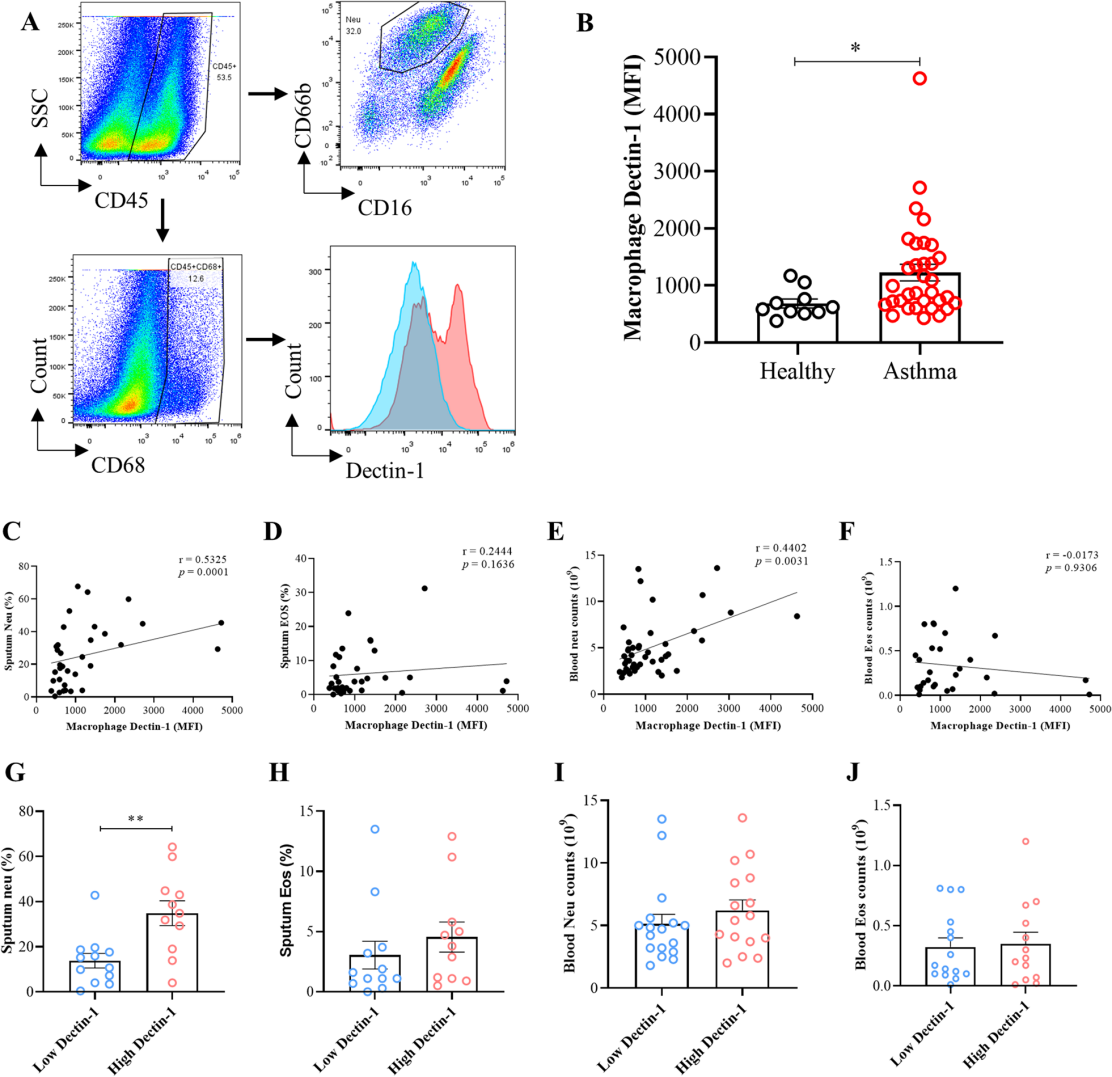
Positive correlation between macrophage Dectin-1 and neutrophils in asthma patients. Induced sputum of 33 asthma patients and 10 healthy controls was collected. **A** Flow gating strategy for macrophages, neutrophils, eosinophils in sputum of asthma patients, and MFI of Dectin-1 on macrophages. **B** Calculation of the MFI of Dectin-1 on induced sputum macrophages of healthy and asthma groups. **C–F** The correlation between MFI of macrophage Dectin-1 and sputum Neu%, sputum EOS%, blood neu count, blood eos count of asthma group. **G–J** Analysis of the difference between low Dectin-1 and high Dectin-1 expression groups of asthma (Sputum Neu%: proportion of neutrophils in induced sputum; sputum EOS%: proportion of eosinophils in induced sputum; blood neu count: peripheral blood neutrophil count; blood eos count: peripheral blood eosinophil count). ( *P < 0.01,**P < 0.05, two-tailed paired Student’s t test, spearman rank correlation, error bars represent mean ± SD)

To verify the potential linkage between macrophage Dectin-1 and neutrophil inflammation in humans, we collected induced sputum samples from asthma patients (n = 33) and healthy control (n = 10) (Additional file 1: Table S1). Utilizing flow cytometry, we detected a marked upregulation of macrophage Dectin-1 expression in induced sputum of asthma patients compared with healthy controls (Fig. 7A and B, p < 0.001). Furthermore, our analysis revealed a positive correlation between the Median fluorescence intensity (MFI) of Dectin-1 and both the proportion of neutrophils in induced sputum (Fig. 7C, r = 0.533, p = 0.001) and the peripheral blood neutrophil count (Fig. 7D, r = 0.440, p = 0.003). Conversely, no significant correlation was observed between Dectin-1 MFI and either the proportion of eosinophils in induced sputum (Fig. 7E, r = 0.244, p = 0.164) or the peripheral blood eosinophil count (Fig. 7F, r = − 0.017, p = 0.931). Collectively, these findings support that macrophage Dectin-1 can influence neutrophils in asthma.

### Positive correlation between macrophage Dectin-1 and caspase-4, IL-1α, IL-1β mRNA expression in asthma patients

**Fig. 8:**
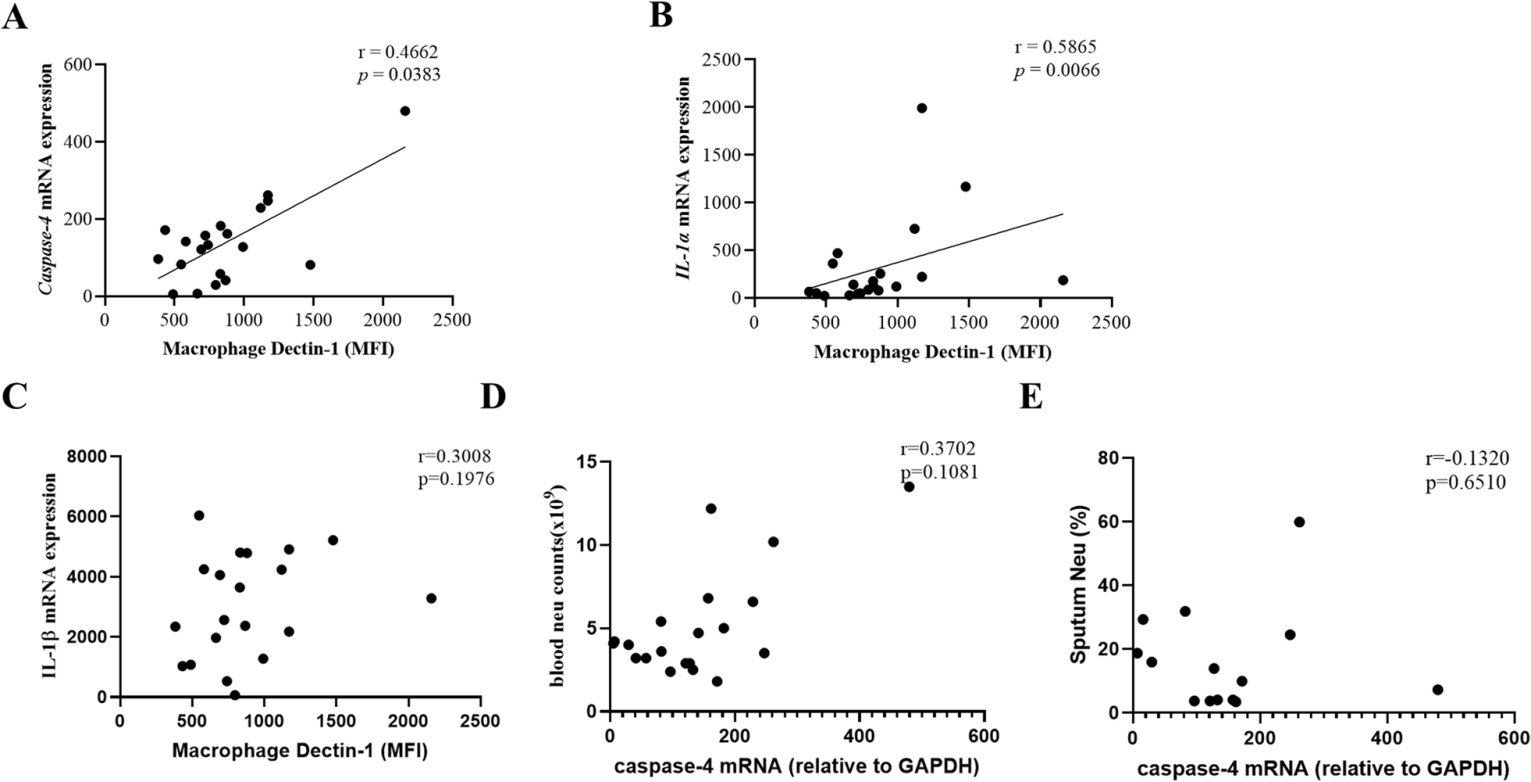
Positive correlation between macrophage Dectin-1 and *caspase-4, IL-1α, IL-1β* mRNA expression in asthma patients. Total RNA of induced sputum from asthma patients was extracted for qRT-PCR detection. **A–C** The correlation between MFI of Dectin-1 on induced sputum macrophages and *caspase-4, IL-1α, IL-1β* mRNA. **D**, **E** The correlation between induced sputum *caspase-4* mRNA and sputum Neu%, blood neu count in induced sputum of asthma patients (Spearman rank correlation)

It is well established that *caspase-4* serves as the human homology of caspase-11 [25]. To investigate the relationship between macrophage Dectin-1 and key pyroptosis markers in asthma patients, RT-qPCR was performed to detect *caspase-4, IL-1α* and *IL-1β* expression in induced sputum. As shown in Fig. 8, macrophage Dectin-1 MFI was positively correlated with the expression of *caspase-4* mRNA (Fig. 8A, r = 0.466, p = 0.038) and *IL-1α* mRNA (Fig. 8B, R = 0.587, P = 0.007), but no correlation with *IL-1β* mRNA (Fig. 8C, r = 0.301, p = 0.198). However, the mRNA expression of *caspase-4* in induced sputum of asthma patients has no significant correlation with peripheral blood neutrophil count (Fig. 8D, r = -0.132, p = 0.651), and the proportion of neutrophils in induced sputum (Fig. 8E, r = 0.370, p = 0.108). Taken together, these results indicate that there is also an association between caspase-4 and macrophage Dectin-1 expression in patients with asthma.

## Discussion

In this study, we have demonstrated that the upregulation of Dectin-1 occurs in HDM-induced asthma mouse model and HDM-treated MH-S cells, leading to the promotion of pyroptosis and neutrophil inflammation. This conclusion is supported by a range of evidences. Specifically, the Dectin-1 agonist Curdlan was able to promote the exacerbation of lung damage in HDM-induced asthmatic mice, primarily characterized by an increase of neutrophils. Conversely, the Dectin-1 inhibitor enable to partially reverse the above changes. In addition, our findings revealed that Curdlan was able to promote activation of caspase-11 and fragmentation of GSDMD in asthmatic mice. Notably, caspase-11 inhibitor can attenuate HDM/Curdlan-induced inflammation, pyroptosis, and neutrophil chemokine levels in asthmatic lung tissues and MH-S cells. Finally, a positive correlation was observed between the expression of macrophage Dectin-1 in induced sputum and the number of neutrophils, as well as pyroptosis related markers in asthmatic patients. Collectively, our research sheds novel insight into the detail molecular mechanisms involving the macrophage Dectin-1 and its critical role in the pathogenesis of asthma.

Dectin-1, a receptor for β-glucan on the cell membrane, playing a pivotal role in antifungal immunity. It facilitates the recruitment of neutrophils through various pathways, including CARD9 [18] and integrin Mac-1 [42]. Recent studies proposed that Dectin-1 is overexpressed in asthma [43, 44], highlighting its potential involvement in asthma pathophysiology. Specifically, the glucan component of asthma allergen HDM can be recognized by Dectin-1 in epithelial cells, leading to the secretion of C–C motif chemokine ligand 20 (CCL20) [45]. And HDM component tropomyosin has also been shown to activate Dectin-1 [21]. However, the specific mechanism and its effect on asthma phenotype are not clear. Our study provides novel insights onto the role of Dectin-1 in asthma. For the first time, we demonstrated that Dectin-1 activation aggravates pulmonary neutrophil inflammation in HDM-induced asthmatic mice. Moreover, we observed the positive correlation between the expression of Dectin-1 in induced sputum macrophages and neutrophils in asthma patients. This correlation may be attributed to the mild activation of Dectin-1 by HDM or potentially due to altered immune inflammatory mechanisms resulting from the interaction between HDM and Dectin-1. Previous studies have found that HDM could be used as an important medium for pro-inflammatory regulatory factors of some membrane proteins [46], thereby promoting more severe airway inflammation. The increased proportion of neutrophils in the airway is often accompanied by more severe late-onset asthma and worsening and persistent airway obstruction [47]. Our findings suggest that Dectin-1 may be a critical mediator of severe neutrophilic asthma, offering a potential therapeutic target for the treatment of this debilitating condition.

In general, Dectin-1 activates phosphatidylinositol 3-kinase (PI3K)/AKT [48], phospholipase C-γ2 (PLC-γ2) and Ca2 + [49] and other signal pathways in spleen tyrosine kinase (syk)-dependent manner. Accumulating evidences have provided that Dectin-1 may be implicated in promoting pyroptosis. For instance, in the model of intracerebral hemorrhage, Dectin-1 signaling pathway upregulates the expression of GSDMD-N, IL-1β and IL-18 in mouse brain cells [32]. Pyroptosis has been shown to effectively recruit neutrophils and inflammatory monocytes to the corresponding sites and play a clear role in bacteria [50]. Notably, widespread lung pyroptosis has been closely related to neutrophilia in inflammatory models such as acute lung injury [51] and influenza [52]. In our study, we found that both HDM and Curdlan stimulation caused a large number of macrophages death and the release of LDH. Further investigations revealed that this process was mediated by caspase-11-dependent cleavage of GSDMD and the subsequent release of IL-1α and IL-1β. Moreover, we also found that the administration of caspase-11 inhibitor wedelolactone significantly reduced pulmonary inflammation in asthma, especially in Dectin-1 activated asthma model. Additionally, the use of wedelolactone reduced pulmonary inflammation in a model of typical neutrophil asthma induced by HDM and LPS. Taken together, these results implicate that macrophage Dectin-1 promotes pulmonary neutrophil inflammation through caspase-11-mediated pyroptosis in asthma.

In our study, caspase-11 inhibitors did not exhibit a significant inhibitory effect on neutrophil count in BALF of asthmatic mice. However, wedelolactone demonstrated an extensive inhibitory effect on pulmonary inflammation in asthma, including overall pulmonary inflammation, goblet cells, eosinophils and lymphocytes in BALF. The possible reason is that the inhibition of caspase-11 by wedelolactone involves changes in complex pathways such as IkappaK/IkappaB/nuclear factor-κB (NF-κB), which may affect other phenotypes of asthma [53]. In summary, wedelolactone emerges as a promising therapeuyic candidate for asthma, not solely limited to neutrophil-dominated asthma. Nevertheless, the precise underlying mechanism warrants further exploration.

Unlike caspase-1, the activation of caspase-11 and its subsequent induction of pyroptosis are highly dependent on lipopolysaccharide (LPS). Caspase-11 can be directly triggered by endotoxin [54], serving as its receptor, or through LPS-activated TLR4 signal transduction [55, 56]. Historically, caspase-11 was mainly considered to be an important medium of infectious diseases [57], but its involvement in non-infectious diseases has been continuously discovered in recent years [30, 58]. However, little is known about the upstream pathway that promotes the activation of caspase-11 in non-infectious diseases. In this study, we provide novel evidence that Dectin-1 may act as an upstream mechanism to activate caspase-11, promoting pyroptosis in asthma. Although Dectin-1 can have a synergistic effect with LPS-activated TLR4 receptors [59, 60], our findings suggest that Dectin-1-mediated caspase-11 activation may occur independent of LPS. This inference is supported by our observation that Dectin-1 inhibitors did not exhibit a notable therapeutic effect in an HDM/LPS induced asthma model. Collectively, these results implicate Dectin-1 as potential upstream regulator of caspase-11 activation and pyroptosis in asthma, independently of LPS influence.

It is well established that Dectin-1/sky can induce the assembly of inflammasome NLRP3 [61, 62], and the activation of caspase-11 is partly dependent on NLRP3 [40]. In attempt to elucidate the intermediary mechanism by which Dectin-1 activates caspase-11, we utilized the NLRP3 inhibitor MCC950 in cells induced with HDM/Curdlan. Notably, we observed a significant reduction in both caspase-11 activation and GSDMD cleavage. These findings suggest that Dectin-1 may activate caspase-11 through NLRP3.

The chemotaxis and infiltration of neutrophils in the airway can be directly mediated by chemokine. CXCL1 is one of the most associated hub genes in the pathogenesis of severe asthma [63], which can be induced by IL-1β, IL-6 and so on [64]. In asthma, the phenotypic changes of macrophages, such as M1 polarization, can promote the pulmonary recruitment of neutrophils through the production of chemokines CXCL1 [65, 66]. Similarly, in renal injury, activation of Dectin-1 promotes the CXCL1-mediated neutrophil migration [67]. Interestingly, the deletion of the caspase-11 gene has been proved to be associated with reduced secretion of CXCL1 in severe acute respiratory syndrome (SARS) [68]. Our study found that Dectin-1/caspase-11 activation in asthma is not only related to CXCL1, but also related to the secretion of CXCL3 and CXCL5. And these chemokines follow the same expression pattern as their shared receptor CXCR2, in the lung tissue of asthma mice. Furthermore, we observed upregulation of IL-17A in the lung of Dectin-1-activated asthma mice, an effect that was suppressed by caspase-11 inhibitors. IL-17A regulates the behavior of neutrophils through cytokines that promote the proliferation and survival of polymorphonuclear cells and neutrophil chemokines (CXCL1, CXCL2 and CXCL5) [69]. Therefore, our study suggested that Dectin-1/caspase-11-mediated macrophage pyroptosis promotes neutrophil airway infiltration in asthma, which depends on the secretion of chemokines such as CXCL1, CXCL3 and CXCL5.

## Conclusion

In summary, the activation of Dectin-1 is intricately linked to neutrophilic inflammation in asthma, specifically by triggering caspase-11-mediated macrophage pyroptosis, which subsequently promotes the secretion of chemokines. Thus, targeting Dectin-1 or caspase-11 through the use of inhibitors or gene knockout techniques may emerge as a promising approach for managing severe asthma.

### Supplementary Information

**Additional file 1: Fig. S1.** The role of simple Curldan and Laminarin in mice. C57BL/6 mice were divided into 4 groups, PBS, PBS + Curdlan and PBS + Laminarin group. Curdlan (20 μg Curdlan in 50 μl PBS) or Laminarin (5 mg/kg, 100 μl) was administered to mice before PBS at days 0, 2, 4, 11 to 14. **(A).** The lung tissue of each group was stained with H&E and PAS (25x). **(B).**The count of total cells and eosinophils, neutrophils, macrophages and lymphocytes in BALF of each mice group by flow cytometry. **Fig. S2.** Wedelolactone had therapeutic effect on neutrophils induced by HDM + LPS in mice. C57BL/6 mice were divided into 4 groups, HDM, HDM + LPS, HDM + LPS + Lam (Laminarin) and HDM + LPS + Wed (wedelolactone) group. 20 μg HDM and 1 μg LPS was instilled intratracheally to mice in 50 μl PBS for three consecutive days (0, 2, 4), then only HDM was given to mice at day 9–12. At day 0, 2, 4, 9–12, the Laminarin or wedelolactone was given before stimulation with HDM or HDM/LPS. **(A).** The mean fluorescence intensity (MFI) of Dectin-1 on inflammatory cells in BALF of mice in each group by flow cytometry. **(B).** The lung tissue of each group was stained with H&E and PAS (25x). **(C-D).** Inflammatory score of lung histopathology by H&E staining and PAS staining. **(E-I).** The count of total cells and eosinophils, lymphocytes, neutrophils and macrophages and in BALF of each mice group by flow cytometry. **Fig. S3.** The expression of caspase-11 in macrophages of HDM or HDM/Curdlan induced mouse lung tissue. Caspase-11 was mainly expressed on macrophages in lung of HDM or HDM/Curdlan-induced mice. **(A).** Representative dual-immunofluorescence staining of Caspase-11 and F4/80 in lung of HDM, HDM/Curdlan-induced mice.

## Data Availability

All other data and materials are included within the article or Additional Information or available from the authors on request.

## Abbreviations

HDM: House dust mite
GSDMD: Gasdermin D
GSDMD-N: N-terminal fragments of gasdermin D
LDH: Lactate dehydrogenase
ICS: Inhaled corticosteroids
PRRs: Pattern recognition receptor
TLRs: Toll-like receptors
NLRs: Nod-like receptors
CARD9: Caspase adaptor recruitment domain family member 9
BCL10: B cell CLL/lymphoma 10
MALT1: MALT1 paracaspase
Th1: T helper 1
Th17: T helper 17
CXCL1: CXC motif chemokine ligand 1
G-CSF: Granulocyte-colony stimulating facto
SAFA: Severe asthma with fungal sensitation
NLRP3: NOD-, LRR- and pyrin domain-containing protein 3
OVA: Ovalbumin
CXCR2: CXC chemokine receptor 2
PBS: Phosphate buffered solution
LPS: Lipopolysaccharide
BALFs: BAL fluids
PAS: Periodic Acid-Schiff
H&E: Hematoxylin and eosin
FBS: Fetal bovine serum
DTT: Dithiothreitol
GSDMD-FL: full length of gasdermin D
iNOS: Inducible nitric oxide synthase
TNF-α: Tumour necrosis factor
MFI: Median fluorescence intensity
CCL20: C-C motif chemokine ligand 20
PI3K: Phosphatidylinositol 3-kinase
PLC- γ2: Phospholipase C-γ 2
NF-κB: Nuclear factor-κB
ROS: Reactive Oxygen Species
syk: Spleen tyrosine kinase
SARS: Severe acute respiratory syndrome
