# Supplementary material for: Dectin-1 aggravates neutrophil inflammation through caspase-11/4-mediated macrophage pyroptosis in asthma
Source: Respir Res. 2024 Mar 8;25:119. doi: 10.1186/s12931-024-02743-z (PMC10921740; doi:10.1186/s12931-024-02743-z)
Supplement: Supplementary file 1 — Additional file 1: Fig. S1. The role of simple Curldan and Laminarin in mice. C57BL/6 mice were divided into 4 groups, PBS, PBS + Curdlan and PBS + Laminarin group. Curdlan (20 μg Curdlan in 50 μl PBS) or Laminarin (5 mg/kg, 100 μl) was administered to mice before PBS at days 0, 2, 4, 11 to 14. (A). The lung tissue of each group was stained with H&E and PAS (25x). (B).The count of total cells and eosinophils, neutrophils, macrophages and lymphocytes in BALF of each mice group by flow cytometry. Fig. S2. Wedelolactone had therapeutic effect on neutrophils induced by HDM + LPS in mice. C57BL/6 mice were divided into 4 groups, HDM, HDM + LPS, HDM + LPS + Lam (Laminarin) and HDM + LPS + Wed (wedelolactone) group. 20 μg HDM and 1 μg LPS was instilled intratracheally to mice in 50 μl PBS for three consecutive days (0, 2, 4), then only HDM was given to mice at day 9–12. At day 0, 2, 4, 9–12, the Laminarin or wedelolactone was given before stimulation with HDM or HDM/LPS. (A). The mean fluorescence intensity (MFI) of Dectin-1 on inflammatory cells in BALF of mice in each group by flow cytometry. (B). The lung tissue of each group was stained with H&E and PAS (25x). (C-D). Inflammatory score of lung histopathology by H&E staining and PAS staining. (E-I). The count of total cells and eosinophils, lymphocytes, neutrophils and macrophages and in BALF of each mice group by flow cytometry. Fig. S3. The expression of caspase-11 in macrophages of HDM or HDM/Curdlan induced mouse lung tissue. Caspase-11 was mainly expressed on macrophages in lung of HDM or HDM/Curdlan-induced mice. (A). Representative dual-immunofluorescence staining of Caspase-11 and F4/80 in lung of HDM, HDM/Curdlan-induced mice. [file 12931_2024_2743_MOESM1_ESM.pdf]

Dectin-1 aggravates neutrophil inflammation through caspase-11/4-mediated macrophage pyroptosis in asthma

Runjin Cai[1,3,*], Xiaoxiao Gong[1,3,*], Xiaozhao Li[2,3], Yuanyuan Jiang[1,3], Shuanglinzi Deng[1,3], Jiale Tang[1,3], Huan Ge[1,3], Chendong Wu[1,3], Huan Tang[1,3], Guo Wang[1,3], Lei Xie[1,3], Xuemei Chen[1,3], Xinyue Hu[1,3,#], Juntao Feng[1,3,#]

[1] Department of Respiratory Medicine, National Key Clinical Specialty, Branch of National Clinical Research Center for Respiratory Disease, Xiangya Hospital, Central South University, Changsha, China; [2] Department of Nephrology, Xiangya Hospital, Central South University, Changsha, China; [3]National Clinical Research Center for Geriatric Disorders, Xiangya Hospital, Central South University, Changsha, China

[*] These authors contributed equally to this work.

[#] Correspondence: Dr. Juntao Feng, Department of Respiratory Medicine, Xiangya Hospital, Central South University, Changsha, Hunan, China. 410008, E-mail: jtfeng1976@csu.edu.cn; Dr. Xinyue Hu, Department of Respiratory Medicine, Xiangya Hospital, Central South University, Changsha, Hunan, China. 410008, E-mail: hxy0215@csu.edu.cn

Table S1 Clinical characteristics of asthma patients in low Dectin-1 expression group and high Dectin-1 expression group

	**No./ Mean**	**Low Dectin-1**	**High Dectin-1**	***P***
**Age**	45.91(12.24)	49.76(13.23)	41.81(9.91)	0.061
**Gender (female)**	20(60.61)	10(58.82)	10(62.50)	0.829
**Age of onset of asthma (y)**	43.61(12.11)	48.24(12.81)	38.69(9.35)	0.021
**Asthma duration (y)**	2.31(3.10)	1.53(2.53)	3.15(3.49)	0.137
**Aggravation per year (times)**				
**0**	7(21.21)	5(29.41)	2(12.50)	0.363
**1**	13(39.39)	7(41.18)	6(37.50)	0.363
**⩾2**	13(39.39)	5(29.41)	8(50.00)	0.363
**Phlegm**				
**Yes**	11(33.33)	7(41.18)	4(25.00)	0.325
**No**	22(66.67)	10(58.82)	12(75.00)	0.325
**FeNO (ppb)**	51.71 (37.77)	54.36(38.33)	48.00(38.69)	0.694
**Lung function**				
**FEV1%**	80.83(16.92)	76.66(18.33)	85.33(14.67)	0.207
**FVC%**	94.77(13.26)	92.09(12.18)	97.67(14.30)	0.303
**FEV1/FVC**	72.08(12.17)	70.50(15.80)	73.80(6.65)	0.499
**Blood neutrophil counts（10^9/L）**	5.645(3.284)	5.116(3.226)	6.206(3.355)	0.349
**Blood eosinophil counts（10^9/L）**	0.355(0.307)	0.353(0.307)	0.358(0.321)	0.966
**Induced sputum**				
**neutrophil %**	23.87(18.18)	13.79(11.16)	34.86(18.30)	0.003
**eosinophil %**	3.76 (4.04)	3.04 (3.98)	4.55(4.15)	0.385

The numerical value is expressed as mean (standard deviation) or number of people (percentage)

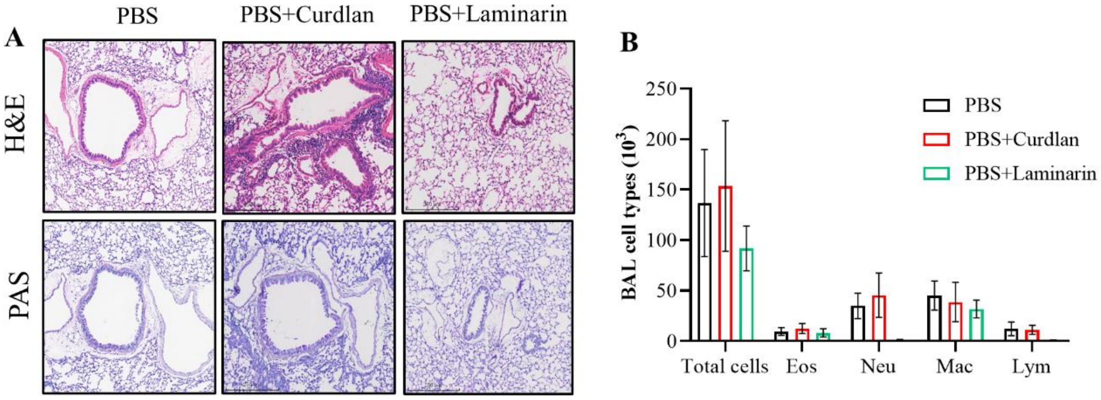

Fig. S1 The role of simple Curdlan and Laminarin in mice.

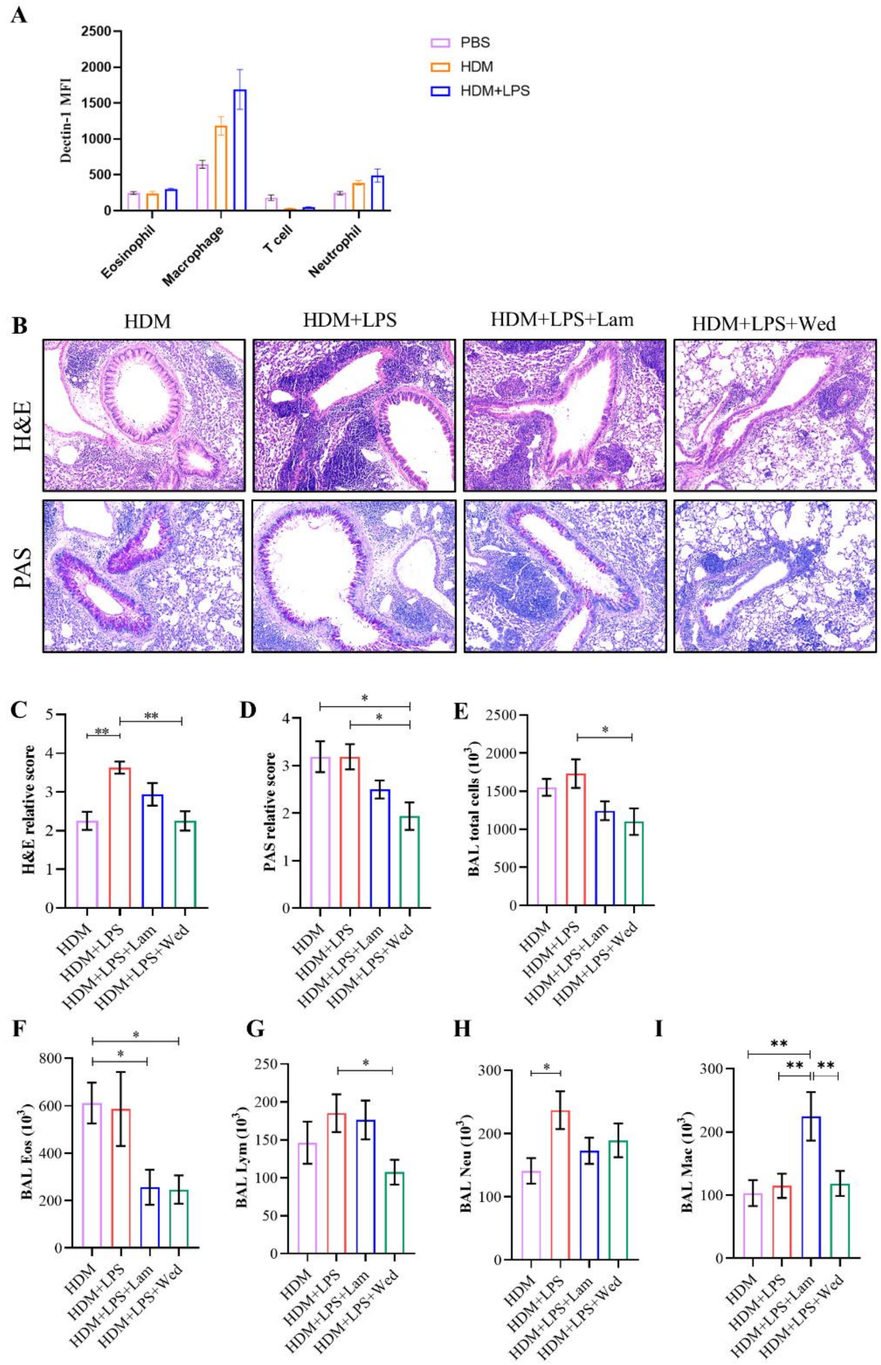

Fig. S2 Wedelolactone had therapeutic effect on neutrophils induced by HDM+LPS in mice

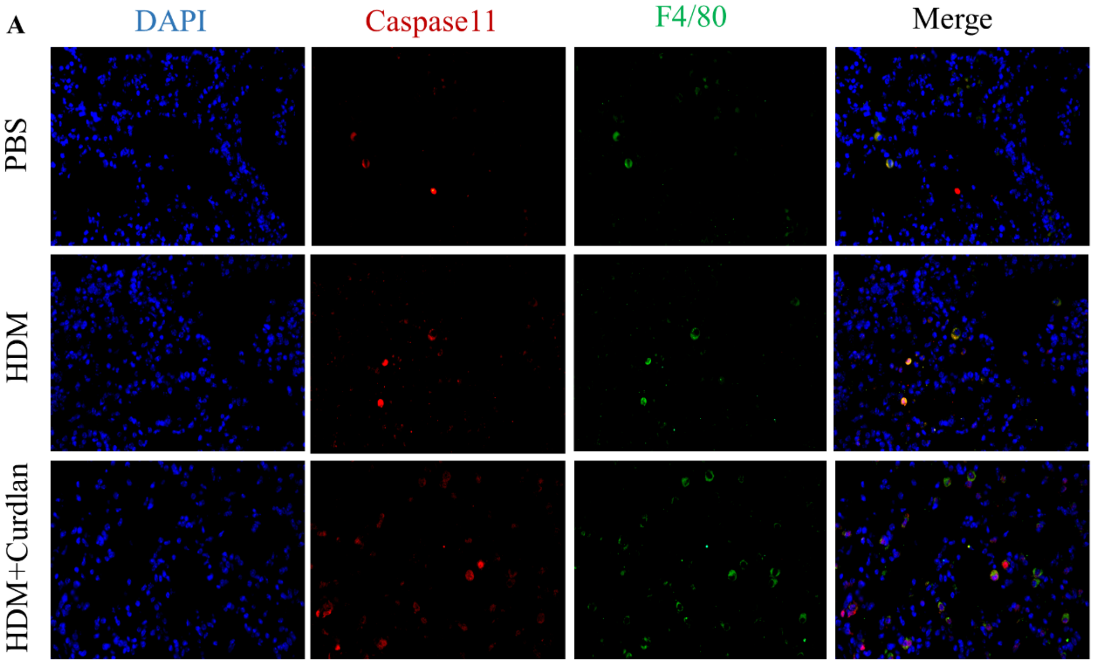

Fig. S3. The expression of caspase-11 in macrophages of HDM or HDM/Curdlan induced mouse lung tissue.
